# Racial and Gender Disparities in the Occurrence and Outcomes of Alcohol-Associated Hepatitis in Hospitalized Patients With Prior Bariatric Surgery

**DOI:** 10.7759/cureus.104251

**Published:** 2026-02-25

**Authors:** Sarpong Boateng, Solomon Gyabaah, Yussif Issaka, Guy Loic Nguefang Tchoukeu, Prince Ameyaw, Chimezirim Ezeano, Yazan Al-Ajlouni, Basile Njei

**Affiliations:** 1 Department of Medicine, Yale New Haven Health System, Bridgeport Hospital, Bridgeport, USA; 2 College of Public Health, University of North Texas, Fort Worth, USA; 3 Department of Internal Medicine, Komfo Anokye Teaching Hospital, Kumasi, GHA; 4 Department of Internal Medicine, Texas Tech University Health Sciences Center, Odessa, USA; 5 School of Medicine, New York Medical College, New York, USA; 6 Department of Medicine, Yale School of Medicine, New Haven, USA

**Keywords:** alcohol-associated hepatitis, bariatric surgery, disparities, metabolic disease, national inpatient sample, obesity, outcomes

## Abstract

Background

There is an increased risk of alcohol use disorder post-bariatric surgery. The impact of bariatric surgery on the outcomes of alcohol-associated hepatitis (AAH) remains unclear. Hence, we aimed to evaluate whether a history of bariatric surgery influences in-hospital outcomes of AAH and whether these associations vary by sex or race.

Methodology

We conducted a retrospective, cross-sectional study using the 2016-2020 National Inpatient Sample to identify adult hospitalizations with AAH and bariatric surgery diagnoses. We performed a 1:1 propensity score matching. Matching variables included baseline characteristics (age, sex, primary payer source, race, etc.), clinical comorbidities (diabetes, hypertension, etc.), and outcomes (in-hospital mortality, acute kidney injury, heart failure, major adverse cardiovascular event, length of stay, death, etc). Multivariable regression was performed to compare in-hospital mortality, hepatic decompensation, and other complications. Subgroup analyses were used to assess disparities by sex and race.

Results

Among 100,910 AAH admissions, 2.3% had a history of bariatric surgery. After matching, bariatric surgery was associated with significantly lower odds of in-hospital mortality (adjusted odds ratio (aOR) = 0.55, 95% confidence interval (CI) = 0.35-0.89), hepatic decompensation (aOR = 0.58, 95% CI = 0.44-0.77), acute kidney injury (aOR = 0.72, 95% CI = 0.60-0.87), and acute respiratory distress syndrome (aOR = 0.64, 95% CI = 0.46-0.91). Female and Black patients had statistically higher likelihood of AAH hospitalization following bariatric surgery compared to males (aOR = 6.90, 95% CI = 6.15-7.74) and White patients (aOR = 1.18, 95% CI = 1.04-1.35), respectively. No differences in outcomes were observed across sex or race.

Conclusions

Among AAH patients, prior bariatric surgery was associated with significantly improved in-hospital outcomes and may suggest a potential protective effect of metabolic optimization.

## Introduction

Alcohol-associated liver disease (ALD) has become a leading cause of liver-related morbidity and mortality in the United States. Within ALD, alcohol-associated hepatitis (AAH) is an acute, life-threatening liver injury triggered by heavy alcohol use, with mortality of up to 30% in severe cases [1]. With a changing epidemiology of AAH, recent analyses report rising hospitalization and mortality rates for ALD, particularly among young adults and women [1]. This trend is driven in part by increased drinking patterns in these demographics [2]. Many patients with AAH have coexisting obesity or metabolic dysfunction, which may exacerbate liver injury through steatosis and inflammation [3]. Importantly, severe AAH can also occur in patients who are malnourished or lean, suggesting that obesity is not a uniform driver of disease severity [4,5]. Bariatric surgery leads to significant and sustained weight loss, thereby reducing comorbidities and mortality in patients with obesity [6], as well as lowering cardiovascular events and improving survival [7]. Additionally, among patients with obesity and compensated cirrhosis, bariatric surgery is associated with improved long-term survival at an acceptable cost, suggesting that improving metabolic health can favorably modify outcomes of chronic liver disease [8].

While bariatric surgery improves metabolic risk factors, it has been associated with an increased risk of alcohol use disorder (AUD) due to a combination of altered alcohol metabolism, changes in neurohormonal and reward pathways, and psychosocial factors such as addiction transfer [6,9]. Post-bariatric surgery, around 20-30% of patients report increased alcohol intake or new AUD, with about 5% developing ALD [10]. Overall, approximately 80% of bariatric procedures in the United States are performed in White individuals, while Hispanic women have the lowest rates [11]. Data on the clinical outcomes of patients who develop severe AAH after surgery are lacking.

Given this background, we designed this study to address two questions: (1) Does a history of bariatric surgery impact the clinical outcomes of patients hospitalized with AAH? (2) Are there gender or racial disparities in the occurrence of AAH and outcomes among patients post-bariatric surgery? By addressing these questions, we aim to inform clinical strategies for risk stratification and long-term follow-up in bariatric patients, while also guiding health policy to improve care for individuals at the intersection of obesity and ALD.

## Materials and methods

Data source and study population

We conducted a retrospective study using the National Inpatient Sample (NIS) database for the years 2016-2020 [12]. The NIS is the largest all-payer inpatient database in the United States, containing a 20% stratified sample of hospital discharges nationwide, and it provides weighted estimates for national hospitalization trends [12].

We identified adult hospitalizations with AAH as the primary diagnosis. The International Classification of Diseases, Tenth Revision, Clinical Modification (ICD-10-CM) diagnostic codes were used to capture AAH, specifically, code K70.1 (alcoholic hepatitis) and its sub-classifications (e.g., K70.11 for alcoholic hepatitis with ascites, K70.10 without ascites). We included discharges aged ≥18 years. We excluded a small number of encounters with missing key outcome data (e.g., missing discharge disposition, which is needed to determine in-hospital mortality) or implausible patient demographics (e.g., invalid age). If a patient had multiple AAH admissions in the dataset, each admission was treated as a separate observation. The entire adult NIS population was used to estimate the odds of AAH hospitalizations. The other analysis was conducted with the AAH-only cohort for assessing outcomes.

Exposure

The exposure of interest was the history of bariatric surgery before or during the index admission. We identified bariatric surgery using a combination of International Classification of Diseases, 10th Revision, Procedure Coding System (ICD-10-PCS) procedure codes and ICD-10-CM diagnosis codes indicative of a past bariatric procedure. The specific codes captured common metabolic/bariatric operations, namely, Roux-en-Y gastric bypass (RYGB), sleeve gastrectomy, biliopancreatic diversion/duodenal switch, and gastric banding. We included codes for both open and laparoscopic approaches. Different bariatric procedures were analyzed as a composite variable.

Outcomes and definitions

The primary outcome was in-hospital mortality, defined by discharge disposition of death in the hospital. Secondary outcomes included hepatic decompensation events, a composite of acute complications of liver failure during hospitalization, including ascites, hepatic encephalopathy, variceal hemorrhage, or hepatorenal syndrome; length of stay (LOS), measured in days from admission to discharge; and hospitalization cost, total hospital charges converted to cost using cost-to-charge ratios, adjusted for inflation to 2020 USD. We also captured patient demographics, hospital characteristics, and comorbid conditions.

Statistical analysis

We first described patient characteristics in the bariatric surgery versus no bariatric surgery groups before matching, including demographics, hospital characteristics, and comorbidities. We compared these characteristics using chi-square tests for categorical variables and t-tests or Wilcoxon rank-sum tests for continuous variables as appropriate.

A propensity score for having undergone bariatric surgery before admission was calculated for each patient using a logistic regression model. A 1:1 matching was performed for the bariatric surgery group versus the non-bariatric surgery group using nearest-neighbor matching without replacement, with a caliper width of 0.2 standard deviations of the logit of the propensity score. Matching variables included baseline characteristics (age, sex, primary payer source, race, median household income, weekend admission, elective admission, region of hospital, hospital size, location), clinical comorbidities (diabetes, hypertension), and outcomes (in-hospital mortality, overall cost, hepatic decompensation, cardiac arrest, sepsis, acute respiratory distress syndrome, gastrointestinal bleeding, acute kidney injury, heart failure, portal vein thrombosis, major adverse cardiovascular event, LOS, death). Balance of covariates post-matching was assessed using standardized mean differences (SMDs), with an SMD <0.1 indicating good balance.

After matching, we compared outcomes between the two groups. Chi-square tests were used for categorical outcomes and t-test or Wilcoxon rank-sum (as appropriate) for continuous outcomes in the unadjusted analysis. We then fit multivariable logistic regression models on the matched to estimate the adjusted odds ratios (aORs) and 95% confidence intervals (CIs) for each binary outcome associated with prior bariatric surgery and AAH. For LOS and cost, we used generalized linear models to estimate adjusted differences. These models adjusted for any residual imbalanced covariates post-propensity score matching.

We subsequently performed subgroup analyses by sex and race. First, we estimated the weighted prevalence of AAH, stratified by sex. We then performed a logistic regression analysis to assess the odds of AAH hospitalization in patients with prior bariatric surgery across race and sex. Finally, we conducted an exploratory analysis to compare outcomes of AAH in patients with prior bariatric surgery by sex and race/ethnicity.

All analyses incorporated NIS survey weights to derive national estimates. A two-sided p-value <0.05 was considered statistically significant. Statistical analyses were performed using SAS 9.4 (SAS Institute Inc., Cary, NC, USA). The study used de-identified public data and was exempt from institutional review board approval.

## Results

Patient characteristics and bariatric surgery utilization

Over the five-year study period (2016-2020), we identified 100,910 adult hospitalizations for AAH among patients in the NIS sample, corresponding to 504,550 weighted hospitalizations nationwide. Among these, only 2.30% had a documented history of bariatric surgery (unweighted n = 2,362). Approximately three-quarters of the AAH patients with prior bariatric surgery were female compared to only about one-third in the no-surgery group (p < 0.01) (Table 1). The cohort’s mean age was around 47.60 ± 12 years, with the majority falling in the 40-60-year age range.

**Table 1:** Baseline characteristics and comorbidities of hospitalized patients with alcohol-associated hepatitis before matching. ^1^: Kruskal-Wallis p-value; ^2^: chi-square p-value.

History of bariatric surgery	No	Yes	Total	Test statistic	P-value
	(N = 98,548)	(N = 2,362)	(N = 100,910)		
Age in years at admission				0.80	0.371
N	98,548	2,362	100,910		
Mean (SD)	47.60 (12.44)	47.40 (9.43)	47.6 (12.38)		
Sex				2,218.25	<0.01^2^
Male	68,421 (69.44%)	563 (23.84%)	68,984 (68.37%)		
Female	30,114 (30.56%)	1,799 (76.16%)	31,913 (31.63%)		
Primary payer source				225.58	<0.01^2^
Private insurance	16,028 (16.30%)	424 (18.00%)	16,452 (16.30%)		
Medicaid	39,411 (40.10%)	774 (32.80%)	40,185 (39.90%)		
Medicare	25,111 (25.50%)	889 (37.70%)	26,000 (25.80%)		
Other payment source	12,803 (13.00%)	185 (7.80%)	12,988 (12.90%)		
Self-pay	1,310 (1.30%)	16 (0.70%)	1,326 (1.30%)		
No charge	3,688 (3.70%)	70 (3.00%)	3,758 (3.70%)		
Race/Ethnicity				82.96	<0.01^2^
White	66,706 (70.00%)	1,680 (73.30%)	68,386 (70.10%)		
Black	10,625 (11.10%)	324 (14.10%)	10,949 (11.20%)		
Hispanic	11,752 (12.30%)	192 (8.40%)	11,944 (12.20%)		
Asian or Pacific Islander	1,189 (1.20%)	12 (0.50%)	1,201 (1.20%)		
Native American	2,309 (2.40%)	20 (0.90%)	2,329 (2.40%)		
Other	2,749 (2.90%)	64 (2.80%)	2,813 (2.90%)		
Median household income ($)				27.60	0.012
<38,999	26,040 (27.30%)	558 (24.10%)	26,598 (27.20%)		
39,000–47,999	24,479 (25.70%)	617 (26.60%)	25,096 (25.70%)		
48,000–62,999	24,572 (25.80%)	609 (26.30%)	25,181 (25.80%)		
>63,000	20,253 (21.20%)	534 (23.00%)	20,787 (21.30%)		
Weekend admission				0.59	0.442
No	73,490 (74.60%)	1,745 (73.90%)	75,235 (74.60%)		
Yes	25,058 (25.40%)	617 (26.10%)	25,675 (25.40%)		
Elective admission				1.56	0.922
No	95,093 (96.60%)	2,278 (96.60%)	97,371 (96.60%)		
Yes	3,336 (3.40%)	79 (3.40%)	3,415 (3.40%)		
Region of the hospital				34.81	<0.01^2^
Northeast	19,659 (19.90%)	557 (23.60%)	20,216 (20.00%)		
Midwest	23,599 (23.90%)	620 (26.20%)	24,219 (24.00%)		
South	28,957 (29.40%)	621 (26.30%)	29,578 (29.30%)		
West	26,333 (26.70%)	564 (23.90%)	26,897 (26.70%)		
Hospital size				0.53	0.762
Small	22,654 (23.00%)	553 (23.40%)	23,207 (23.00%)		
Medium	30,149 (30.60%)	707 (29.90%)	30,856 (30.60%)		
Large	45,745 (46.40%)	1,102 (46.70%)	46,847 (46.40%)		
Location/Teaching status				8.04	0.022
Rural	7,377 (7.50%)	142 (6.00%)	7,519 (7.50%)		
Urban non-teaching	22,588 (22.90%)	532 (22.50%)	23,120 (22.90%)		
Urban teaching	68,583 (69.60%)	1,688 (71.50%)	70,271 (69.60%)		
Elixhauser Comorbidity Index				54.18	<0.01^1^
N	76,145	1,804	77,949		
Mean (SD)	5.20 (1.75)	5.50 (1.77)	5.20 (1.75)		
Diabetes				13.62	<0.01^2^
No	91,946 (93.30%)	2,249 (95.20%)	94,195 (93.30%)		
Yes	6,602 (6.70%)	113 (4.80%)	6,715 (6.70%)		
Hypertension				2.09	0.152
No	54,056 (54.90%)	1,331 (56.40%)	55,387 (54.90%)		
Yes	44,492 (45.10%)	1,031 (43.60%)	45,523 (45.10%)		
Obesity				1,165.63	<0.01^2^
No	88,911 (90.20%)	1,621 (68.60%)	90,532 (89.70%)		
Yes	9,637 (9.80%)	741 (31.40%)	10,378 (10.30%)		

Significant differences in race and ethnicity were observed between groups (p < 0.01) (Table 1). Patients with prior bariatric surgery were more likely to be non-Hispanic White (around 73% of the surgery group vs. 70% of the no-surgery group) and less likely to be Hispanic (8% vs. 12% in the no-surgery group). The proportion of Black patients was similar between groups (about 14% in the surgery group vs. 11% in the no-surgery group). Other racial groups (Asian/Pacific Islander, Native American, and other/unknown) each constituted 1-3% of the cohort in either group. Socioeconomic differences were also evident in the primary payer mix (p < 0.01). Patients with prior bariatric surgery were more likely to have private insurance (18% vs. 16) and less likely to be insured through Medicaid (33% vs. 40%) compared to those without bariatric surgery.

Table 2 presents the baseline characteristics after 1:1 propensity score matching. We matched 2,242 patients with a history of bariatric surgery to 2,242 patients without bariatric surgery. Post-matching balance diagnostics demonstrated excellent covariate balance, with SMDs ranging from 0.000 to 0.042 across key variables, including age, calendar year, comorbidity burden, socioeconomic status, and hospital characteristics. The range of SMDs was well below the conventional threshold of 0.10, indicating excellent balance between groups (Table 2).

**Table 2:** Baseline characteristics and comorbidities of hospitalized patients with alcohol-associated hepatitis after 1:1 propensity-matched analysis. ^1^: Kruskal-Wallis p-value; ^2^: chi-square p-value.

Variable			P-value	Test statistic	Standardized difference
History of bariatric surgery	No	Yes			
	(N = 2,242)	(N = 2,242)			
Age in years at admission			0.45^1^	0.57	0.01
N	2,242	2,242			
Mean (SD)	47.20 (12.24)	47.30 (9.43)			
Sex			0.17^2^	1.88	0.04
Male	500 (22.30%)	539 (24.04%)			
Female	1,742 (77.70%)	1,703 (75.56%)			
Primary payer source			0.99^2^	0.30	0.04
Private Insurance	398 (17.80%)	403 (18.00%)			
Medicaid	728 (32.50%)	735 (32.80%)			
Medicare	851 (38.00%)	841 (37.50%)			
Other payment source	183 (8.20%)	179 (8.00%)			
Self-pay	14 (0.60%)	16 (0.70%)			
No charge	68 (3.00%)	68 (3.00%)			
Race/Ethnicity			0.66^2^	3.25	0.12
White	1,665 (74.30%)	1,644 (73.30%)			
Black	305 (13.60%)	319 (14.20%)			
Hispanic	192 (8.60%)	186 (8.30%)			
Asian or Pacific Islander	14 (0.60%)	12 (0.50%)			
Native American	11 (0.50%)	19 (0.80%)			
Other	55 (2.50%)	62 (2.80%)			
Median household income ($)			0.57^2^	2.01	0.07
<38,999	571 (25.50%)	538 (24.00%)			
39,000–47,999	599 (26.70%)	602 (26.90%)			
48,000–62,999	586 (26.10%)	585 (26.10%)			
>63,000	486 (21.70%)	517 (23.10%)			
Weekend admission			0.54^2^	0.38	0.04
No	1,669 (74.40%)	1,651 (73.60%)			
Yes	573 (25.60%)	591 (26.40%)			
Elective admission			0.33^2^	0.98	0.02
No	2,181 (97.30%)	2,170 (96.80%)			
Yes	61 (2.70%)	72 (3.20%)			
Region of the hospital			0.93^2^	0.18	0.11
Northeast	544 (24.30%)	546 (24.40%)			
Midwest	573 (25.60%)	568 (25.30%)			
South	583 (26.00%)	600 (26.80%)			
West	542 (24.20%)	528 (23.60%)			
Hospital size			0.62^2^	0.95	0.00
Small	500 (22.30%)	525 (23.40%)			
Medium	707 (31.50%)	685 (30.60%)			
Large	1,035 (46.20%)	1,032 (46.00%)			
Location/Teaching status			0.51^2^	1.35	0.06
Rural	131 (5.80%)	130 (5.80%)			
Urban non-teaching	471 (21.00%)	503 (22.40%)			
Urban teaching	1,640 (73.10%)	1,609 (71.80%)			
Elixhauser Comorbidity Index			0.03^2^	4.62	0.00
N	1,705	1,706			
Mean (SD)	5.4 (1.83)	5.5 (1.77)			
Diabetes			0.35^2^	0.87	0.015
No	2,117 (94.40%)	2,131 (95.00%)			
Yes	125 (5.60%)	111 (5.00%)			
Hypertension			0.76^2^	0.00	0.01
No	1,246 (55.60%)	1,256 (56.00%)			
Yes	996 (44.40%)	986 (44.00%)			
Obesity			1.00^2^	0.00	0.00
No	1,530 (68.20%)	1,530 (68.20%)			
Yes	712 (31.80%)	712 (31.80%)			

In-hospital outcomes: mortality and complications

**Figure 1:**
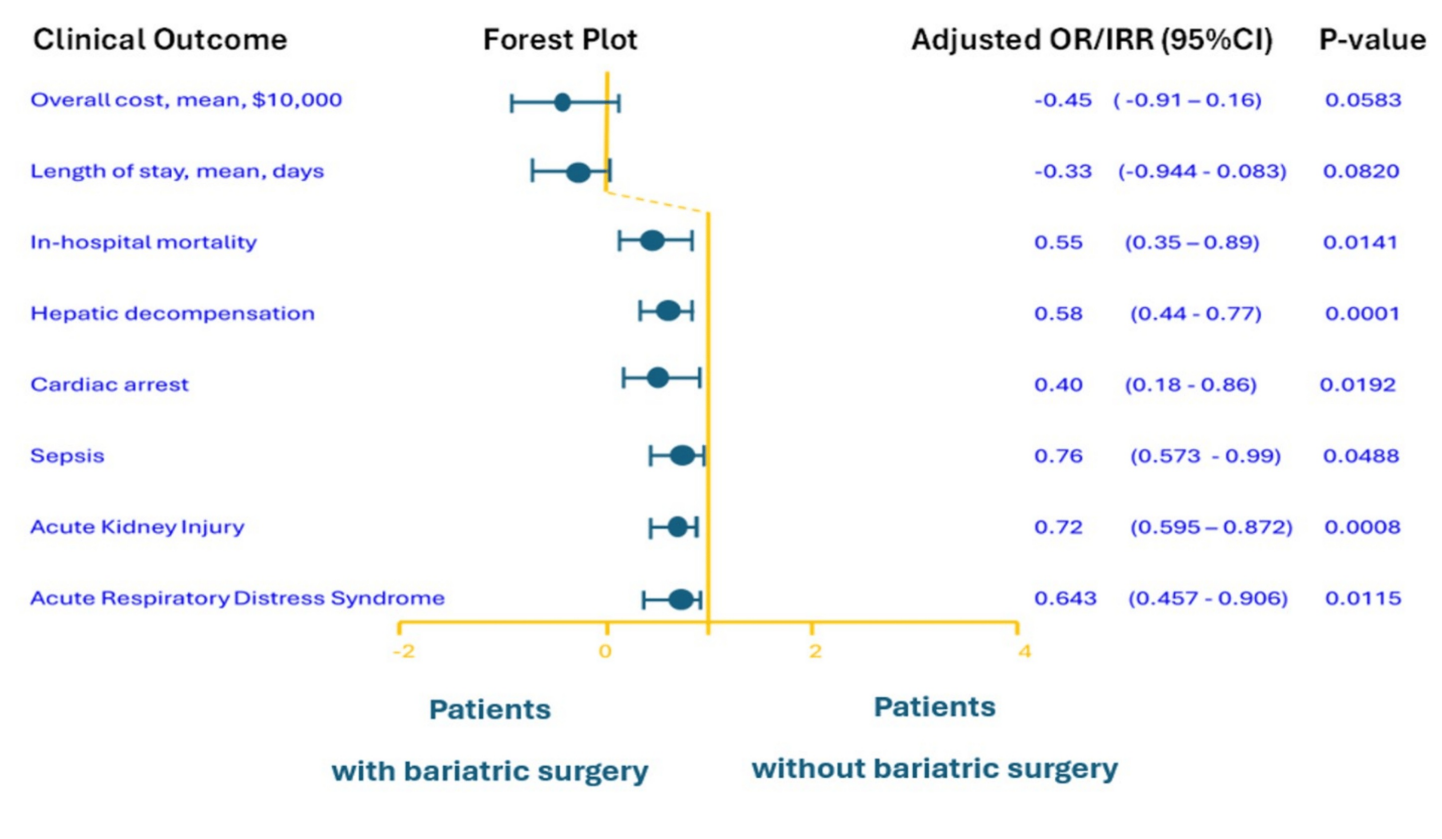
Adjusted ratios (ORs or IRRs) and 95% confidence intervals (CIs) for key clinical outcomes comparing hospitalized alcohol-associated hepatitis patients with versus without a history of bariatric surgery. Bariatric surgery was associated with reduced odds of in-hospital mortality and major complications.

After matching, patients with prior bariatric surgery had a 45% lower odds of in-hospital mortality from AAH compared to patients without prior bariatric surgery (aOR = 0.55, 95% CI = 0.35-0.89; p = 0.01) (Figure 1).

We also observed significant reductions in several major complications of AAH among patients with prior bariatric surgery (Figure 1). In the matched cohort, the incidence of acute hepatic decompensation events was lower in the bariatric surgery group. Specifically, the adjusted odds of any hepatic decompensation were 42% lower in the bariatric group compared to the no-surgery group (aOR = 0.58, 95% CI = 0.44-0.77; p = 0.01) Similarly, there was a 28% reduction in the odds of acute kidney injury among patients with prior bariatric surgery (aOR = 0.72, 95% CI = 0.60-0.87; p < 0.01). Other complications followed a similar trend in the adjusted model with significantly lower odds observed among patients with a history of bariatric surgery: cardiac arrest (aOR = 0.40, 95% CI = 0.18-0.86; p = 0.02), acute respiratory distress syndrome (aOR = 0.64, 95% CI = 0.46-0.91; p = 0.01), and sepsis (aOR = 0.76, 95% CI = 0.58-0.99; p = 0.04). The odds of gastrointestinal bleeding, portal vein thrombosis, and shock were comparable between the bariatric and non-bariatric groups, with no significant differences: gastrointestinal bleeding (aOR = 1.18, 95% CI = 0.89-1.57; p = 0.26), portal vein thrombosis (aOR = 0.74, 95% CI = 0.30-1.84; p = 0.52), and shock (aOR = 0.87, 95% CI = 0.53-1.42; p = 0.57) (Table 3).

**Table 3:** Association between prior bariatric surgery and outcomes of alcohol-associated hepatitis. Outcomes assessed using multivariable logistic regression (binary outcomes). Adjusted models controlled for age group, race, sex, and Elixhauser comorbidity index. The Wald test statistic was conducted, and chi-square values are provided. ACS = acute coronary syndrome; AKI = acute kidney injury; MI = myocardial infarction; PE = pulmonary embolism; DVT = deep vein thrombosis; GI = gastrointestinal; ARDS = acute respiratory distress syndrome; MACE = major adverse cardiovascular events; OR = odds ratio; aOR = adjusted odds ratio; CI = confidence interval

Alcohol-associated hepatitis outcomes (bariatric surgery vs. no bariatric surgery)	OR	95% CI	Test statistics	P-value	aOR	95% CI	Test statistics	P-value
Cardiac arrest	0.38	0.19–0.76	7.57	<0.01	0.40	0.18–0.86	5.53	0.02
ACS	0.21	0.08–0.57	9.63	<0.01	0.24	0.07–0.80	8.12	0.02
AKI	0.71	0.60–0.83	18.3	<0.01	0.72	0.60–0.87	11.45	<0.01
Sepsis	0.84	0.66–1.07	1.97	0.16	0.76	0.57–0.99	4.46	0.05
Ileus	0.37	0.19–0.72	8.48	<0.01	0.45	0.21–0.95	4.52	0.03
Acute MI	0.25	0.09–0.66	7.73	0.01	0.7	0.08–0.92	5.96	0.04
Shock	0.81	0.53–1.24	0.93	0.33	0.87	0.53–1.42	0.23	0.57
Venous thrombosis	0.48	0.25–0.91	5.06	0.02	0.58	0.28–1.22	1.94	0.15
PE	1.00	0.53–1.89	0.00	1.00	1.15	0.55–2.44	0.11	0.71
DVT	1.00	0.42–2.41	0.00	1.00	1.10	0.40–3.08	1.94	0.85
Portal vein thrombosis	0.59	0.27–1.28	1.76	0.18	0.74	0.30–1.84	0.34	0.52
GI bleeding	1.12	0.88–1.44	0.88	0.35	1.18	0.89–1.57	2.08	0.26
Decompensated liver disease	0.67	0.57–0.86	11.35	<0.01	0.58	0.44–0.77	6.33	<0.01
Abdominal CT	1.06	0.54–2.11	0.03	0.86	1.36	0.62–3.01	0.70	0.44
ARDS	0.62	0.46–0.83	9.94	<0.02	0.64	0.46–0.91	6.65	0.01
Paracentesis	1.51	0.80–2.84	1.59	0.21	1.27	0.61–2.63	0.81	0.52
Cholecystectomy	2.35	1.07–5.13	4.55	0.03	1.85	0.77–4.42	1.90	0.17
Cholangiogram	2.01	0.75–5.35	1.93	0.16	1.73	0.57–5.25	0.99	0.33
Parenteral nutrition (TPN)	1.51	0.53–4.23	0.59	0.44	1.10	0.33–3.70	0.02	0.87
MACE	0.75	0.62–0.90	9.87	<0.01	1.13	0.89–1.42	0.03	0.32
Heart failure	0.87	0.69–1.10	1.30	0.25	1.29	0.93–1.79	0.20	0.12
Death	0.64	0.43–0.99	4.78	0.03	0.55	0.35–0.89	6.33	0.01

Length of stay and resource utilization

The median LOS in the bariatric surgery group was 7 days (interquartile range (IQR) = 4-12 days) compared to 8 days (IQR = 5-14) for the no-surgery group. In the adjusted analysis, this difference was not statistically significant (adjusted mean difference of about -0.33 days, 95% CI = -0.65 to +0.01, p = 0.05).

Hospitalization costs were high for AAH in general. The mean total cost per admission was about $85,000-$90,000 (2020 USD) in both groups. After adjusting for factors, the average cost for the bariatric group was about $4,500 less than for those without prior surgery (-$4,477, 95% CI = -$9,112 to +$158), with a p-value of 0.06 (Table 4).

**Table 4:** Association between prior bariatric surgery outcomes of alcohol-associated hepatitis and length of stay and resource utilization. Outcomes were assessed using generalized linear regression (continuous outcomes). IRR = incidence rate ratio; CI = confidence interval

Alcohol-associated hepatitis outcomes (bariatric surgery vs. no bariatric surgery)	IRR	95% CI	Test statistics	P-value
Inflation-adjusted charges, ($)	-4,569.23	-8,839.38 to -299.09	4.40	0.04
Inflation-adjusted charges, ($)	-4,477.17	-9,112.13 to 157.78	4.80	0.06
Length of stay (days)	-0.12	-0.42 to 0.18	0.60	0.44
Length of stay (days)	-0.33	-0.65 to 0.01	3.81	0.05

Subgroup analyses by sex and race

The weighted prevalence of AAH hospitalizations was significantly lower among patients with a history of bariatric surgery compared to those without. Of the 504,550 weighted hospitalizations for AAH, only 2.34% (n = 11,810) occurred in patients with prior bariatric surgery, while 97.66% (n = 492,740) occurred in patients without a history of bariatric surgery (Table 5).

**Table 5:** Weighted prevalence of alcohol-associated hepatitis hospitalizations in patients with and without a history of bariatric surgery (2016-2020) Weighted estimates were calculated using national sampling weights from the National Inpatient Sample (NIS) 2016–2020. CIs are based on survey-weighted estimates. Values represent U.S. hospitalizations for alcohol-associated hepatitis. CI = confidence interval

	Overall		History of bariatric surgery		No history of bariatric surgery	
Characteristics	Weighted number of U.S. hospitalizations, 2016–2020	% (95% CI)	Weighted number of U.S. hospitalizations, 2016–2020	Prevalence, % (95% CI)	Weighted number of U.S. hospitalizations, 2016–2020	Prevalence, % (95% CI)
Sex						
Male	344,920	68.37 (68.06–68.68)	2,815	0.82 (0.75–0.89)	342,105	99.18 (99.11–99.25)
Female	159,565	31.63 (31.32–31.94)	8,995	5.64 (5.37–5.91)	159,565	94.36 (94.09–94.63)
Total	504,550	100	11,810	2.34 (2.24–2.44)	492,740	97.66 (97.56–97.76)

Stratified by sex, the prevalence of AAH hospitalizations in males was generally higher (68.37%, 344,920 weighted hospitalizations) compared to females (31.63%, 159,565 weighted hospitalizations). However, among patients with prior bariatric surgery, the prevalence of AAH was markedly lower in males (0.82%) than in females (5.64%). Logistic regression analysis revealed that sex and race significantly modified the odds of AAH hospitalization in patients with prior bariatric surgery. Females had a substantially higher odds of hospitalization for AAH compared to males (aOR = 6.90, 95% CI = 6.15-7.74, p < 0.01) (Tables 6, 7).

**Table 6:** Association between race and sex with hospitalization and alcohol-associated hepatitis in patients with prior bariatric surgery. Outcomes were assessed using multivariable logistic regression. Adjusted models controlled for age group, race, sex, Elixhauser Comorbidity Index, insurance type, hospital bed size, admission type, and location/teaching status. aOR = adjusted odds ratio; OR = odds ratio; CI = confidence interval; ref= reference

Variable		Unadjusted			Adjusted		
		OR (95% CI)	Test statistic	P-value	aOR (95% CI)	Test statistic	P-value
Gender	Females vs. males	7.26 (6.60–7.99)	22,406.96	<0.01	6.90 (6.15–7.74)	1,073.74	<0.01
Race	Whites (Ref)						
	Blacks	1.21 (1.07-1.37)	8.98	<0.01	1.18 (1.04–1.35)	7.08	<0.01
	Hispanics	0.65 (0.56-0.75)	33.0	0.58	0.80 (0.68–0.93)	7.82	0.32
	Asians	0.40 (0.23-0.71)	10.3	0.03	0.56 (0.32–1.00)	3.96	0.30
	Native American/Pacific Indians	0.92 (0.72-1.19)	0.42	0.10	1.08 (0.83–1.40)	0.34	0.20

**Table 7:** Association of bariatric surgery and alcohol-associated hepatitis hospitalization strongly modified by gender. Outcomes were assessed using multivariable logistic regression. Adjusted models controlled for age group, race, sex, Elixhauser Comorbidity Index, insurance type, hospital bed size, admission type, and location/teaching status. Stratified analysis examined the interaction between sex and bariatric surgery on the odds of hospitalization with alcohol-associated hepatitis. aOR = adjusted odds ratio; OR = odds ratio; CI = confidence interval

Outcome		OR (95% CI)	P-value	Test statistic	aOR (95% CI)	P-value	Test statistic
Alcoholic hepatitis							
All patients	Females versus males	0.72 (0.62–0.94)	<0.01	9.57	0.94 (0.88–1.07)	0.10	1.54
Patients with bariatric surgery	Females versus males	7.26 (6.60–7.99)	<0.01	1,653.21	6.90 (6.15–7.74)	<0.01	1,084.19

Regarding race, the odds of AAH hospitalization were higher for Black patients (aOR = 1.18, 95% CI = 1.04-1.35, p < 0.01), while Hispanic (aOR = 0.80, 95% CI = 0.68-0.93, p = 0.32) and Asian (aOR = 0.56, 95% CI = 0.32-1.00, p = 0.30) patients had lower odds compared to Whites (reference group) (Table 5).

We further examined clinical outcomes among AAH patients with a history of bariatric surgery, stratified by sex and race/ethnicity. Outcomes evaluated included major adverse cardiovascular events, acute kidney injury, sepsis, decompensated liver disease, and in-hospital mortality. No significant differences in outcomes were observed across sex or racial/ethnic groups.

## Discussion

In this large national analysis of hospitalized AAH patients, we found striking disparities in the occurrence of AAH after bariatric surgery: women and Black patients were disproportionately represented among post-surgical AAH hospitalizations. Importantly, despite these disparities in occurrence, we also observed that hospitalized patients with AAH and prior bariatric surgery had significantly better inpatient outcomes compared to those without such a history, including lower mortality and reduced odds of hepatic decompensation, acute kidney injury, respiratory failure, sepsis, and cardiac arrest.

While prior studies have explored the risk of ALD following bariatric surgery [9,13], this study is among the first to highlight racial and gender disparities in AAH hospitalizations among this population. This study is also the first national study to challenge prevailing assumptions that prior bariatric surgery worsens alcohol-related liver outcomes and suggests a potentially protective association during acute AAH episodes.

Previous studies have emphasized the potential harms of bariatric surgery in patients with heavy alcohol use. For example, a recent single-center study of 2,634 patients hospitalized for AAH reported that those with prior RYGB had no difference in inpatient mortality but did have higher 30-day readmission rates and significantly worse longer-term outcomes, including increased cirrhosis progression and post-discharge mortality [14]. An earlier NIS-based analysis also reported that prior RYGB was associated with increased risk of hepatic encephalopathy and serious infections among patients hospitalized with ALD, although in-hospital mortality did not differ [15]. These earlier reports align with the established concern that bariatric surgery, especially RYGB, can exacerbate alcohol’s hepatotoxic effects [16,17]. However, our study provides a new perspective: in the acute hospital phase of AAH, prior bariatric surgery was not associated with worse outcomes, and, in fact, was linked to significantly better short-term outcomes. Several factors may explain this divergence. Unlike the single-center RYGB study, our analysis included all types of bariatric procedures (RYGB, sleeve gastrectomy, etc.) and employed propensity score matching to reduce confounding. Our cohort is more contemporary (2016-2020) and reflects a shift in surgical trends, with sleeve gastrectomy now more common and potentially less impactful on alcohol metabolism than RYGB [17]. Finally, by restricting our cohort to patients with obesity-related history, we ensured both groups shared a similar metabolic background, which may have minimized “healthy survivor” bias and allowed us to isolate differences attributable to bariatric surgery itself.

Several hypotheses could explain the observed protective association of prior bariatric surgery with acute AAH outcomes. First, patients who underwent bariatric surgery likely achieved substantial weight loss and metabolic improvement before their AAH episode. Weight loss leads to reduced visceral adiposity, lower systemic inflammation, and improved insulin sensitivity, all of which could positively influence the course of critical illnesses such as severe AAH [18-20]. In contrast, patients with no prior surgery who present with AAH and obesity may suffer a “double hit,” i.e., the synergistic liver insult of obesity-related metabolic dysfunction plus alcohol toxicity [21]. Prior bariatric surgery might attenuate one component of this synergy by ameliorating the obesity and metabolic syndrome factors [18]. It is plausible that in patients with a history of obesity and heavy alcohol use, weight loss, whether surgical or not, reduces baseline hepatic steatosis/fibrosis and systemic inflammation, such that when AAH occurs, the liver and other organs are able to tolerate acute injury. This may explain the significantly lower odds of complications such as acute kidney injury and acute respiratory distress syndrome observed in the bariatric group.

Another contributing factor may be selection and healthcare engagement. Patients who undergo bariatric surgery typically have extensive pre-surgical evaluation and post-surgical follow-up [22,23]. Those who developed AAH despite prior bariatric surgery may represent a subgroup with better healthcare access, potentially leading to earlier recognition and management of complications. Further, it may be that patients with prior bariatric surgery experience a less severe form of AAH (perhaps requiring lower alcohol exposure to trigger hepatitis due to altered alcohol kinetics) and thus present with lower baseline liver injury severity. Unfortunately, granular data on AAH severity (e.g., bilirubin or creatinine levels) were not available in our dataset. Nevertheless, the significantly reduced occurrence of acute-on-chronic liver failure features, captured in our composite “hepatic decompensation” outcome, among patients with prior bariatric surgery suggests they may have presented with less advanced chronic liver disease. In essence, these patients might develop AAH earlier in their disease trajectory, potentially leading to better survival prospects. This interpretation is supported by reports showing that RYGB patients have an elevated risk of developing ALD, including AAH, which may indicate a “fast-tracked” progression to severe injury without prolonged subclinical disease [6,24]. Post-bariatric surgery patients experience better management of any ensuing metabolic syndrome, including lower rates of obesity and diabetes at the time of acute liver injury, which reduces the severity of the initial presentation and reduces the adverse outcome of AAH [9]. Furthermore, higher household income and lower-level education have been identified as risk factors for AUD following bariatric surgery. However, in our study, we did not observe any significant association in the household income of the patients between those who have had prior bariatric surgery and those who have not had bariatric surgery. In addition, patients with previous bariatric surgery are well represented in addiction treatment programs, suggesting that they may be more likely to seek, or be referred to, treatment for their alcohol use disorder [13,25].

Our findings have several important clinical implications. First, they underscore the necessity for proactive alcohol use screening and counseling in high-risk patients undergoing bariatric surgery. Bariatric programs should ensure long-term vigilance for hazardous drinking, especially among female and Black patients. Early intervention may prevent progression to AAH. Second, our results deliver a cautiously optimistic clinical message: an episode of AAH post-bariatric surgery does not confer worse immediate prognosis; indeed, it may be associated with a better short-term outcome compared to non-surgical peers. Clinicians can be somewhat reassured that a history of bariatric surgery does not increase in-hospital mortality risk. Nevertheless, management of AAH remains complex; these patients still require robust initiation of secondary prevention strategies, such as alcohol cessation support and referral to addiction services, because prior studies have demonstrated that post-bariatric surgery patients are at an elevated risk for progressive liver disease over time, particularly among women [26,27].

We acknowledge several limitations of our study. Foremost, the retrospective observational design using NIS is subject to unmeasured confounding and coding inaccuracies. We relied on ICD-10 codes to identify AAH, comorbidities, and prior bariatric surgery status, making misclassification a possibility. We attempted to capture bariatric surgery history comprehensively using both procedure codes and status codes, but we could not determine the timing of surgery relative to the AAH hospitalization or the amount of weight lost. Another limitation is the absence of clinical details, such as laboratory values, in the NIS. Hence, we could not directly compare the severity of AAH between groups. We partly mitigated this by matching on comorbidity burden. Furthermore, there is a chance of selection bias as patients who undergo surgery are screened for psychosocial stability and health. Post-bariatric surgery patients are more likely to be enrolled in an AUD treatment program. Despite these limitations, our study boasts notable strengths. To our knowledge, it is the first nationally representative analysis examining racial and gender disparity outcomes of AAH in the context of prior bariatric surgery, leveraging a large sample size with contemporary ICD-10 coding. The use of propensity score matching improved the comparability of groups, and the consistency of findings across multiple outcomes supports the robustness of the association.

## Conclusions

This study demonstrated that among patients with an obesity-related history hospitalized for AAH, those with prior bariatric surgery experience significantly improved in-hospital outcomes, including lower mortality and complication rates. However, this clinical benefit is offset by disparities in the demographic profile of those affected. Women and Black patients were overrepresented among post-bariatric AAH hospitalizations, suggesting disproportionate vulnerability in these groups. These findings highlight the importance of integrating metabolic and addiction care while optimizing postoperative care and long-term monitoring.
